# Human CD27+ memory B cells colonize a superficial follicular zone in the palatine tonsils with similarities to the spleen. A multicolor immunofluorescence study of lymphoid tissue

**DOI:** 10.1371/journal.pone.0229778

**Published:** 2020-03-18

**Authors:** Marie Lettau, Annika Wiedemann, Eva Vanessa Schrezenmeier, Claudia Giesecke-Thiel, Thomas Dörner

**Affiliations:** 1 Department of Rheumatology and Clinical Immunology, Charité University Medicine Berlin, Berlin, Germany; 2 German Rheumatism Research Center Berlin (DRFZ), Berlin, Germany; 3 Department of Nephrology and Medical Intensive Care, Charité University Medicine Berlin, Berlin, Germany; 4 Department of Rheumatology and Clinical Immunology, Formerly at the Charité University Medicine Berlin, Berlin, Germany; 5 Max Planck Institute for Molecular Genetics, Berlin, Germany; Monash University, AUSTRALIA

## Abstract

**Background:**

Memory B cell (mBC) induction and maintenance is one of the keys to long-term protective humoral immunity. MBCs are fundamental to successful medical interventions such as vaccinations and therapy in autoimmunity. However, their lifestyle and anatomic residence remain enigmatic in humans. Extrapolation from animal studies serves as a conceptual basis but might be misleading due to major anatomical distinctions between species.

**Methods and findings:**

Multicolor immunofluorescence stainings on fixed and unfixed frozen tissue sections were established using primary antibodies coupled to haptens and secondary signal amplification. The simultaneous detection of five different fluorescence signals enabled the localization and characterization of human CD27+CD20+Ki67- mBCs for the first time within one section using laser scanning microscopy. As a result, human tonsillar mBCs were initially identified within their complex microenvironment and their relative location to naïve B cells, plasma cells and T cells could be directly studied and compared to the human splenic mBC niche. In all investigated tonsils (n = 15), mBCs appeared to be not only located in a so far subepithelial defined area but were also follicle associated with a previous undescribed gradual decline towards the follicular mantle comparable to human spleen. However, mBC areas around secondary follicles with large germinal centers (GCs) in tonsils showed interruptions and a general widening towards the epithelium while in spleen the mBC-containing marginal zones (MZ) around smaller GCs were relatively broad and symmetrical. Considerably fewer IgM+IgD+/- pre-switch compared to IgA+ or IgG+ post-switch mBCs were detected in tonsils in contrast to spleen.

**Conclusions:**

This study extends existing insights into the anatomic residence of human mBCs showing structural similarities of the superficial follicular area in human spleen and tonsil. Our data support the debate of renaming the human splenic MZ to ‘superficial zone‘ in order to be aware of the differences in rodents and, moreover, to consider this term equally for the human palatine tonsil.

## Introduction

Long-lived memory lymphocytes are key to lifelong immunity against encountered pathogens. Their activation and differentiation takes place in secondary lymphatic organs (SLOs), which in humans include the lymph nodes, the spleen as well as the mucosa-associated lymphatic tissues (MALT) such as tonsils. One special feature of the organization of all SLOs is that the T and B lymphocytes are largely segregated into different anatomical compartments, a process shown to be directed to a great extent by chemokines and adhesion molecules. [1] The paracortex of lymph nodes, the periarterial lymphatic sheath (PALS) of the spleen, and the interfollicular region (IFR) of MALTs designate the organ-specific T cell localization zones. In all SLOs, the term lymph follicle, henceforth referred to as follicle, is the designation of spherical B cell aggregates. From rodent studies, primary follicles are considered to be survival-supporting environments for recirculating naïve B cells, the latter which proliferate in response to T cell dependent antigen and undergo affinity maturation during the germinal center (GC) reaction in so termed secondary follicles. [2] The resulting memory B cells (mBCs) are, next to antibody secreting plasma cells (PCs), major mediators of our humoral immunological memory, since they induce an immediate effector response upon antigen re-encounter. Note, most successful vaccines are based on induction and long-term survival of both, mBCs and PCs. Yet, while the survival niche and survival factors of long-lived PCs were vigorously investigated and largely unraveled [3, 4], only little is known in this regard about mBCs.

A large proportion of human mBCs appears to recirculate, however, the majority of human mBCs resides within lymphatic tissues and share many phenotypic and clonal similarities. [5, 6] Organ-specific mBC niches and their potential survival and maturation characteristics remain, however, unclear. This lack of knowledge is striking with regard to the designation of the human splenic follicular mantle (FM)-surrounding mBC compartment as human marginal zone (MZ). While it is still common to extrapolate from the rodent MZ, there are multiple hints that besides anatomical differences, the majority of rodent organ-specific MZ B cells differ from human mBCs in terms of their function, mutation state as well as migratory behavior. [7–11] Moreover, most human immune tissue studies describe unspecific cellular architectures based on hematoxylin and eosin or single antigenic characterization with immunohistochemistry of human spleen sections compared to multicolor immunofluorescence studies in mice. [10] Human subepithelial regions (SE) of MALTs and the inner wall of the subcapsular sinus in lymph nodes are regarded as mBC compartments and potential human MZ equivalents. [7, 12] Studies indicate a more widespread follicle surrounding mBC distribution [13, 14] but here as well, detailed multicolor immunofluorescence studies are lacking for the majority of different anatomic localizations.

This study aimed to extend the existing investigations in humans to delineate the residence of mBCs by establishing and applying five-color immunofluorescence stainings on frozen tissue sections of palatine tonsil and spleen. The complex staining enabled unambiguous identification of resting mBCs by labelling them as CD27+CD20+Ki67- cells. We found that mBCs colonized a superficial follicular zone in both spleen and tonsil, formerly referred to as human MZ. Consistently, the data support a nomenclature change of tissue resident mBC structures from “MZ-like” towards ‘superficial zone’ as proposed before [9, 11], which is applicable for human spleen and tonsils.

## Materials and methods

### Specimens

Initially, seven spleen specimens S1-S7 (Table 1) served for the establishment of the new staining technology, while these samples did not carry characteristic secondary follicles. Interestingly, all sections from immunotrombocytopenia spleens exclusively contained small follicles, partly with densely packed material within their center. Specimens characterized by small follicles already attracted attention by Steiniger [15], who refers to central follicular amyloid-like hyaline material.

**Table 1:** Spleen and tonsil specimens used in the current study including underlying diagnosis and age of the donors.

No.	Age [years] / sex	Diagnosis
Spleen specimens		
S1	67 / unknown	Tumor nes
S2	45 / female	Splenomegaly and pancytopenia
S3	43 / female	Immunotrombocytopenia
S3	70 / unknown	Immunotrombocytopenia
S5	51 / unknown	Immunotrombocytopenia
S6	Unknown / unknown	Immunotrombocytopenia
S7	20 / unknown	Immunotrombocytopenia
S8	16 / female	Cysts nes
S9	79 / female	Metastatic intestinal tumor nes
Tonsil specimens		
T1	3 / female	Tonsillitis nes
T2	26 / female	Tonsillitis nes
T3	5 / male	Tonsillitis nes
T4	22 / female	Tonsillitis nes
T5	46 / male	Tonsillitis nes
T6	33 / male	Tonsillitis nes
T7	40 / male	Tonsillitis nes
T8	34 / female	Tonsillitis nes
T9	20 / female	Tonsillitis nes
T10	Unknown / unknown	Unknown
T11	Unknown / unknown	Unknown
T12	Unknown / unknown	Unknown
T13	Unknown / unknown	Unknown
T14	Unknown / unknown	Unknown
T15	Unknown / unknown	Unknown

Nes = not elsewhere specified.

Of particular note, the samples S8 and S9 (Table 1), obtained from two splenectomies without an underlying autoimmune disease, exhibited several secondary follicles in the common tissue structure to which the following localization studies refer. All palatine tonsil specimens (n = 15, Table 1) showed huge GCs for mBC localization evaluation.

Written informed consent was obtained in all cases, in accordance with the local ethics’ committee of the Charité University Medicine Berlin (EA1/303/12).

### Tissue preservation

Fresh 1 cm^3^ native tissue samples were briefly immersed in PBS to wash off blood residue, then rested for 5 min in excess Tissue-Tek O.C.T. Compound (Sakura Finetek, Alphen aan den Rijn, Netherlands), transferred to a Tissue-Tek cryomold (Sakura Finetek) and subsequently frozen in liquid nitrogen for 30 sec and stored at -80°C until tissue sections were prepared.

### Immunofluorescence multicolor staining for confocal laser scanning microscope (LSM)

Each tissue was cut into 8 μm sections using a freezing microtome. Sections were placed onto SuperFrost Plus Slides (Thermo Scientific, Waltham, MA, USA) and dried at room temperature (RT) for 30 min before subsequent usage or storage at -80°C.

For immunofluorescence staining, sections were blocked for 5 min with 1x PBS buffer / 3% BSA (Sigma-Aldrich, St. Louis, MO, USA). After careful washing in PBS, sections were incubated with primary antibodies (Table 2, pre-fixation and post-fixation antibody usage as indicated) in PBS / 0.5% BSA for 20 min on a 300 rpm platform shaker in the dark at RT, followed by careful washing. Sections were then incubated with secondary antibodies again for 20 min as performed for primary stainings. After careful washing, the sections were fixed in pre-cooled (-20°C) acetone (Carl Roth, Karlsruhe, Germany) at -20°C for 5 min. This approach was found to provide clearest signals for staining with primary mouse antibodies. Staining with primary rabbit antibodies and secondary antibodies was then performed accordingly after fixation. After embedding with Mounting Medium containing 4,6-Diamidine-2-Phenylindole (DAPI) (Dako, Glostrup, Denmark), covered slides were stored in the dark at 4°C overnight before microscopic evaluation.

**Table 2:** Primary and secondary antibodies used by the current study for multicolor immmunofluorescence microscopy.

	Conjugate	Clone	Isotype	Manufacturer	Acetone fixation
Primary Abs					
Anti-IgA	FITC	M24A	Mouse IgG1	Chemicon	After staining
Anti-IgD	BIO	IA6-2	Mouse IgG2a	BD Pharmingen	After staining
	PE	IA6-2	Mouse IgG2a	BD Pharmingen	After staining
	FITC	IA6-2	Mouse IgG2a	BD Pharmingen	After staining
Anti-IgG	BIO	G18-145	Mouse IgG1a	BD Pharmingen	After staining
Anti-IgM	Pure	Polyclonal	Rabbit	DAKO	Before staining
Anti-κ	BIO	G20-193	Mouse IgG1a	BD Pharmingen	After staining
Anti-λ	BIO	JDC-12	Mouse IgG1a	BD Pharmingen	After staining
Anti-CD3	DIG	UCHT1	Mouse IgG1	DRFZ	After staining
	PE	UCHT1	Mouse IgG1	DRFZ	After staining
Anti-CD27	FITC	M-T271	Mouse IgG1	BD Pharmingen	After staining
	PE	M-T271	Mouse IgG1	BD Pharmingen	After staining
Anti-CD20	pure	Polyclonal	Rabbit	Thermo Scientific	Before staining
Anti-Ki67	PE-Cy7	B65	Mouse IgG1	BD Pharmingen	After staining
	FITC	MIB-1	Mouse IgG1	DAKO	After staining
Anti-MAdCAM-1	DIG	314G8	Mouse IgG1	Abcam	After staining
Secondary Abs					
SA	AF594			Vector Laboratories	
	AF 633			Invitrogen	
Anti-DIG	AF647			DRFZ	
Anti-Rabbit	AF594		Donkey	Jackson Immunoresearch	
	AF647		Donkey	Jackson Immunoresearch	
	FITC		Donkey	Jackson Immunoresearch	
Anti-FITC	AF488			Life Technologies	
Anti-PE	TRITC			Acris	

Ab = antibody.

Conjugates: FITC = fluorescein isothiocyanate, BIO = biotin, PE = phycoerythrin, DIG = digoxigenin, PE-Cy7 = phycoerythrin-cyanin7, SA = Streptavidin, AF = Alexa Fluor, TRITC = tetramethylrhodamine.

Manufacturer: Chemicon, Tenecula, CA, USA; BD Pharmingen, Franklin Lakes, NJ, USA; DAKO, Glostrup, Denmark; German Rheumatism Research Center Berlin (DRFZ), Berlin, Germany; Thermo Scientific, Waltham, MA, USA; Abcam, Cambridge, UK; Vector Laboratories, Burlingame, CA, USA; Invitrogen, Carlsbad, CA, USA; Jackson Immunoresearch, Cambridge, UK; Life Technologies, Carlsbad, CA, USA; Acris, Herford, Germany.

We used a confocal LSM (LSM710, Carl Zeiss, Oberkochen, Germany) for five-color fluorescence detection (Table 3) with the corresponding software 'ZEN system 2010' (Carl Zeiss) and 'ZEN 2012 lite blue edition' software (Carl Zeiss) for image analysis, processing and interpretation, i.e. fluorescence intensitiy measurements.

**Table 3:** Distribution of LSM channels and dyes of the corresponding detection antibodies.

Channel 1	Channel 2	Channel 3	Channel 4 + Channel 5	
DAPI	Mouse-Ab-ITC	Mouse-Ab-PE/PEcy7	Mouse-Ab-DIG	Primary Abs
			Mouse-Ab-BIO	
			Ab (rabbit)	
	Anti-FITC-AF488	Anti-PE-TRITC	Anti-DIG-AF594/647	Secondary Abs
			SA-AF594/633	
			Donkey-anti-rabbit-AF594/647	

The five LSM channels used are arranged in ascending order of the fluorescence wavelength ranges with assignment of the Abs listed in Table 2. Ab = antibody.

## Results

### Definition of human mBCs for histological detection

**Fig 1:**
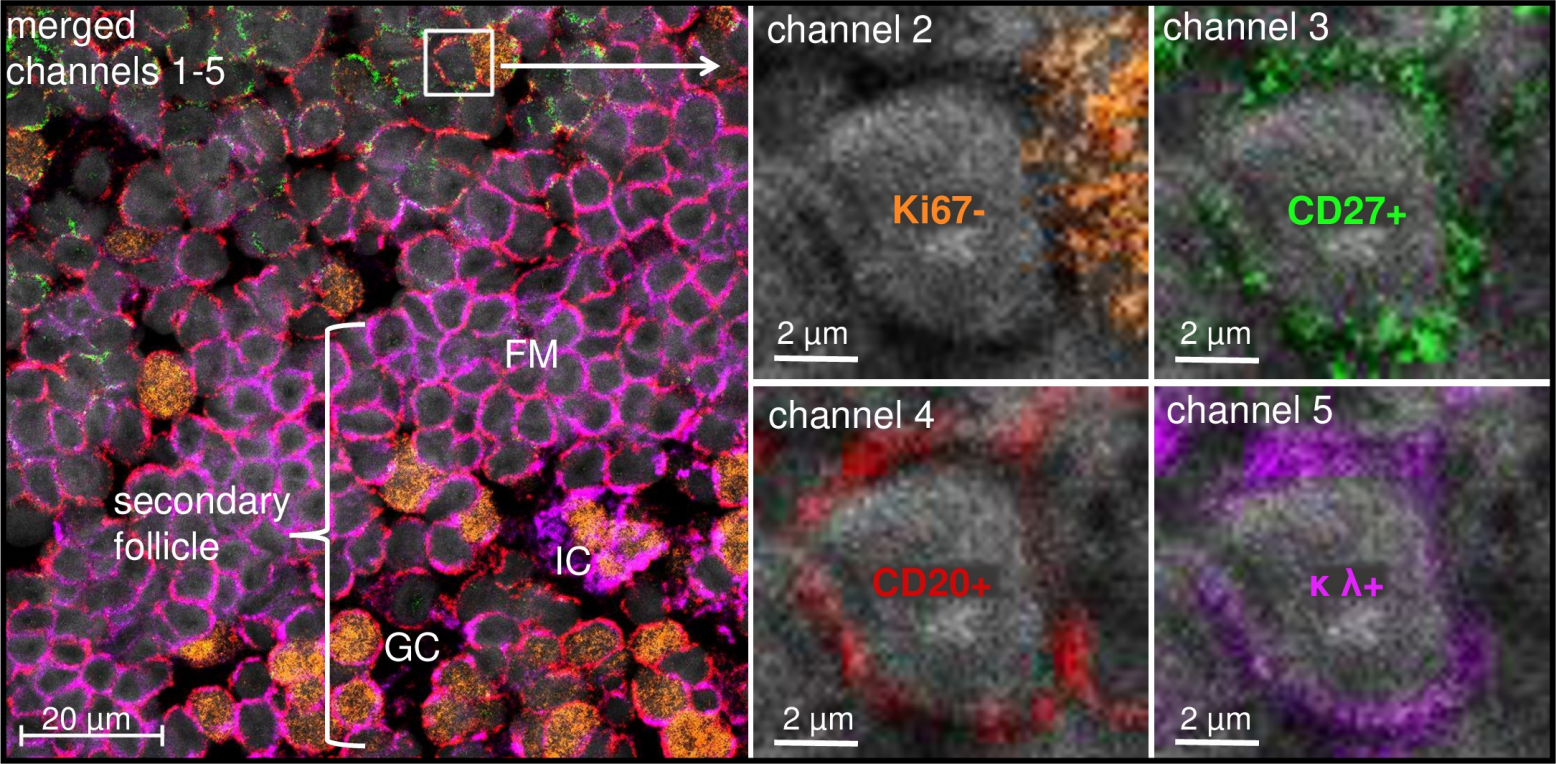
Histological identification of mBCs. [human tonsil; 63x objective]. Human mBCs (single mBC exemplified in white box; localized near a secondary follicle portion) were defined as resting (Ki67- / channel 2 (fluorochrome a)), antigen-experienced (CD27+ / channel 3 (fluorochrome b)) B cells (CD20+ / channel 4 (fluorochrome c) and DAPI / channel 1). The additional channel 5 (fluorochrome d) serves for further characterization (κλ as example for mBC isotype characterization). Within the GC, intercellular signals may arise from immune complexes (IC) on the resident follicular dendritic cells.

For reliable histological detection, we defined mBCs as resting, antigen-experienced B cells as described previously [5, 16]. Based on this definition, four fluorescence channels were required for their identification (Fig 1): DAPI for cell identification (channel 1), Ki67 as an exclusion marker for proliferating cells (channel 2), CD27 as mBC marker (channel 3) [16, 17], that is, however, also expressed by CD3+ T cells and CD20low/- PCs and CD20 (channel 4) as a B cell specific surface antigen. Using the additional channel 5, mBCs or their surrounding cells were further characterized.

### Histological identification and characterization of human splenic white pulp mBCs

Initial detection of human splenic MZ mBCs served as method verification and validation. The following evaluations refer to secondary follicles, containing GC, and PALS in the white pulp of spleen samples identified in S8 and S9. We excluded follicles without definable GC, which was the case for all follicles of samples S1 –S7. Of note, classical primary follicles containing only naïve B cells were not found in any of the examined sections of the samples S1 –S9.

**Fig 2:**
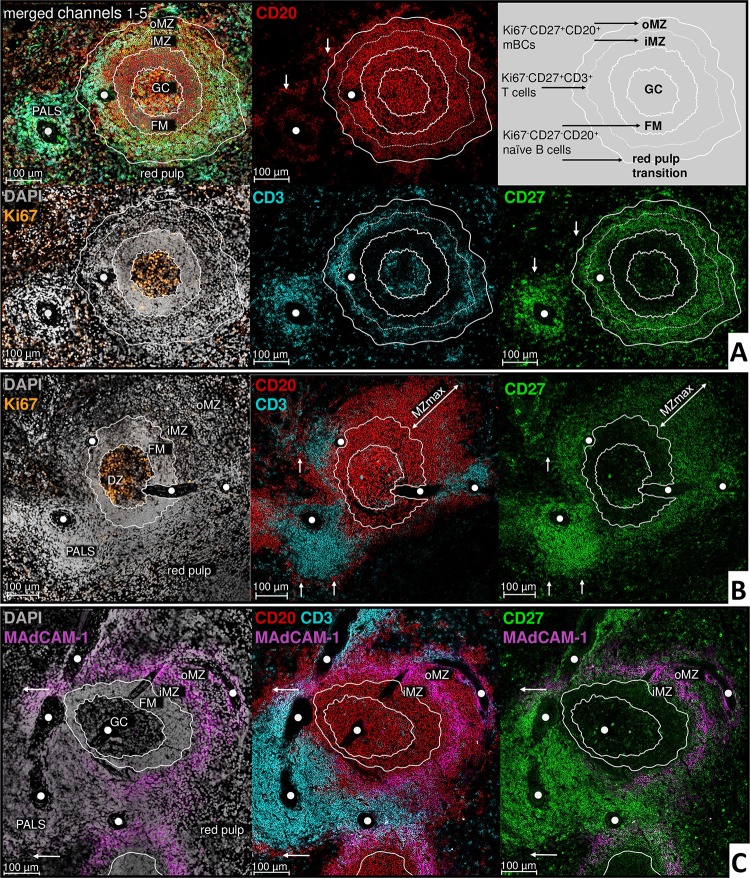
Human splenic symmetrical and asymmetrical secondary follicles. [A-C: sections of human spleen (A = S8; B/C = S9); 20x objective]. White lines are drawn within and around the follicle for better orientation of follicular zones in the different channels. Main population of inner MZ (iMZ) and outer MZ (oMZ) are Ki67-CD27+CD20+ mBCs. At the MZ, instead of a clear border, mBC numbers are decreasing towards the red pulp and the FM. White arrows: Ki67-CD27-CD20+ naïve B cells in the red pulp transition. White circles: Branches of central artery. **A: Representative example of a symmetrical secondary follicle.** Ki67-CD27++CD3+ T cells appear lined-up in the middle of the MZ. Ki67+ cells are largely limited to the GC with no clear light zone (LZ) and dark zone (DZ) polarization. **B: Asymmetrical secondary follicle.** Polarization of the GC with Ki67 rich DZ orientation towards the PALS; MZ widening with increasing distance from the PALS (MZmax). **C: MZ subdivision by MAdCAM-1.** MAdCAM-1 expressed in a ring-like fashion splitting the MZ into an inner part (iMZ) directed to the GC and a MZ outer part (oMZ) directed to the red pulp.

In the white pulp of human spleen, Ki67-CD27-CD20+CD3- naïve B cells, Ki67-CD27+CD20+CD3- mBCs and Ki67-CD27++CD20-CD3+ T cells could be identified (Fig 2). In accordance with flow cytometry data, CD27 expression was stronger on T cells compared to mBCs. Except for few cells in the GCs, we did not find Ki67-CD27+++CD20-CD3- PCs located in the white pulp of the sections analyzed (n>30) but in the red pulp as reported before. [9]

Ki67+ proliferating cells were largely limited to the GC itself, where they were distributed homogeneously over the entire GC. Although we cannot assess how this arrangement was influenced by cutting of individual sections, we detected many circular GCs (n>30) with such non-polarized distribution. We henceforth termed such secondary follicles ‘symmetrical’ (Fig 2A). In contrast, we identified only some ‘asymmetrical’ secondary follicles (visually observed in n = 5) characterized by a more elliptical GC with a distinguishable light zone (LZ) and a Ki67-rich dark zone (DZ), the latter directed to the PALS areas (Fig 2B). In all other parts of the white pulp, Ki67+ cells were largely absent.

The main population in the area surrounding the GC and FM, referred to as the MZ, were mBCs. Correlating with identification of symmetrical secondary follicles were T cells, which appeared organized in a band within the MZ center (Fig 2A). This T cell ring was variable in size ranging from one to several cells per row, partly with interruptions. In contrast, few sections (visually observed in n = 3) contained individual T cells scattered throughout the entire MZ. Of note, we repeatedly found mucosal addressin cell adhesion molecule 1 (MAdCAM-1) expressed also in a ring-like fashion within all sections, clearly dividing the MZ into an inner part (iMZ) directed to the GC and an outer part (oMZ) directed towards the red pulp (Fig 2C). The MZ showed irregular borders, characterized by a decline of mBCs towards the red pulp in the oMZ and towards the FM in the iMZ. For asymmetrical secondary follicles, the MZ widened with increasing distance from the PALS (Fig 2B).

**Fig 3:**
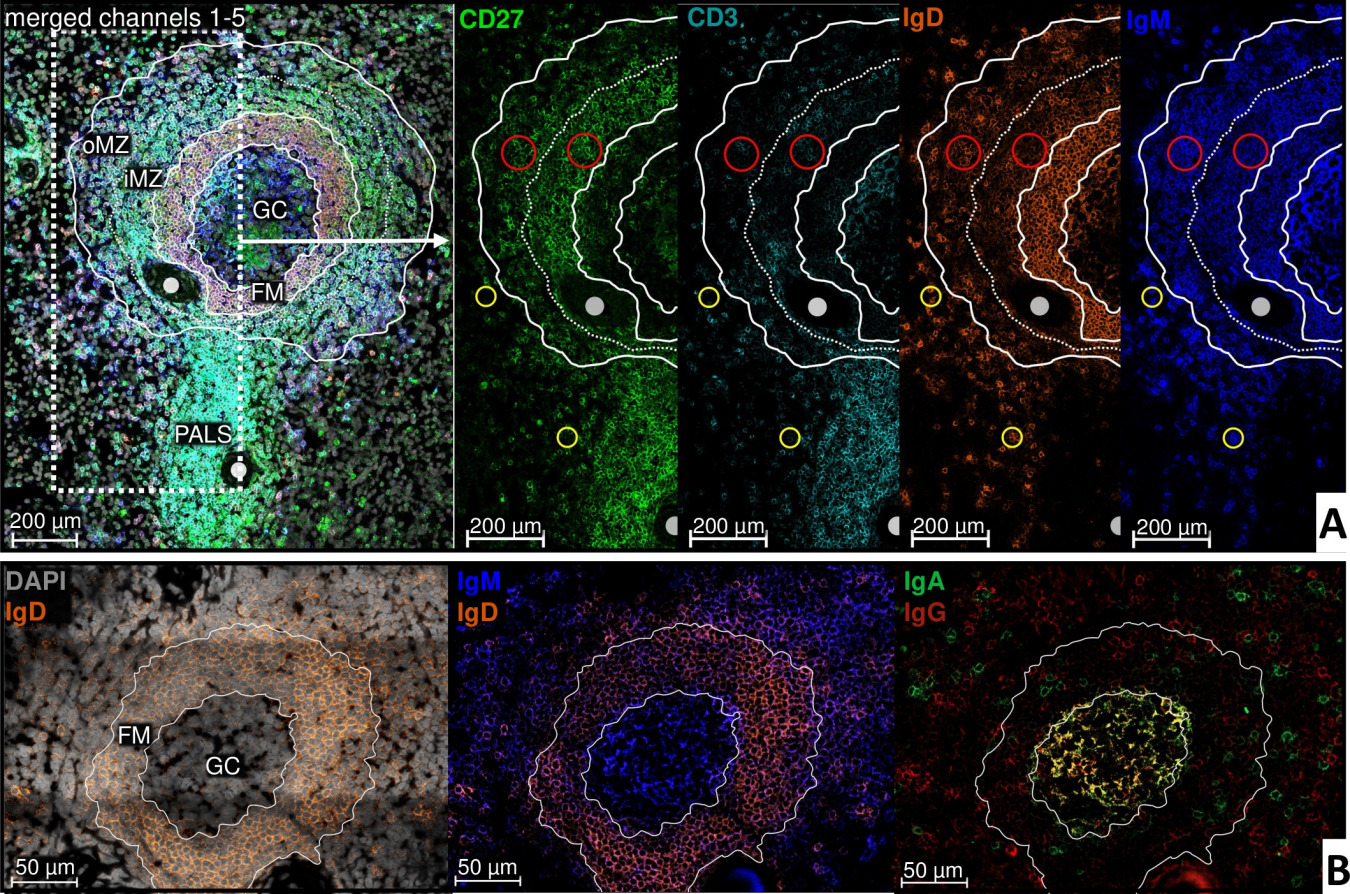
Class switched splenic mBCs are heterogeneously distributed in the superficial zone around the secondary follicle with few mBCs in the FM. **A** [human spleen (S8); 20x objective]: White dashed lines within and around the follicle for better orientation of follicular zones in the different channels. The majority of MZ CD27+CD3- mBCs strongly expresses IgM, in the iMZ together with mostly only low or no surface IgD, in the oMZ mainly together with IgD (compare red circles). The number of IgM-IgD- mBCs increases in direction to the red pulp. CD27-CD3- naïve B cells in the red pulp transition show an increased signal for IgM and IgD (exemplified in yellow circles). White circles: Branches of central artery. **B** [human spleen (S8), 63x objective]: Heterogeneous distribution of IgG+ and IgA+ cells in the MZ and FM. Immunocomplexes in the GC.

In the iMZ, the majority of mBCs expressed IgM with low and partially no surface IgD, whereas oMZ mBCs largely co-expressed IgM with IgD (Fig 3A). The density of IgM-IgD- post-switch mBCs increased towards the direction to the red pulp but was generally less frequent compared to IgM+IgD+ mBCs. Additional stainings for IgG+ or IgA+ demonstrated heterogeneously distributed post-switch mBCs scattered throughout the entire MZ (Fig 3B) individually or in accumulations of few cells without a predominant isotype and with the tendency to increase towards the red pulp. Additionally, individual IgG+ or IgA+ mBC were also identified in the FM.

Naïve B cells preferentially populated densely within the FM. Notably, they were also detected at the oMZ red pulp transition with a more loose organization and increased IgM and IgD expression (Fig 3A). They partially surrounded the entire PALS (Fig 2A–2C) as well as the central artery. Otherwise, B cells were only sporadically found within the PALS.

In summary, the established stainings enabled a detailed assessment of mBC organization within the human MZ. This follicle associated MZ could be subdivided into an oMZ and an iMZ with a mixed population of mBCs gradually declinig towards the follicular mantle.

### Histological identification and characterization of human tonsillar mBCs

**Fig 4:**
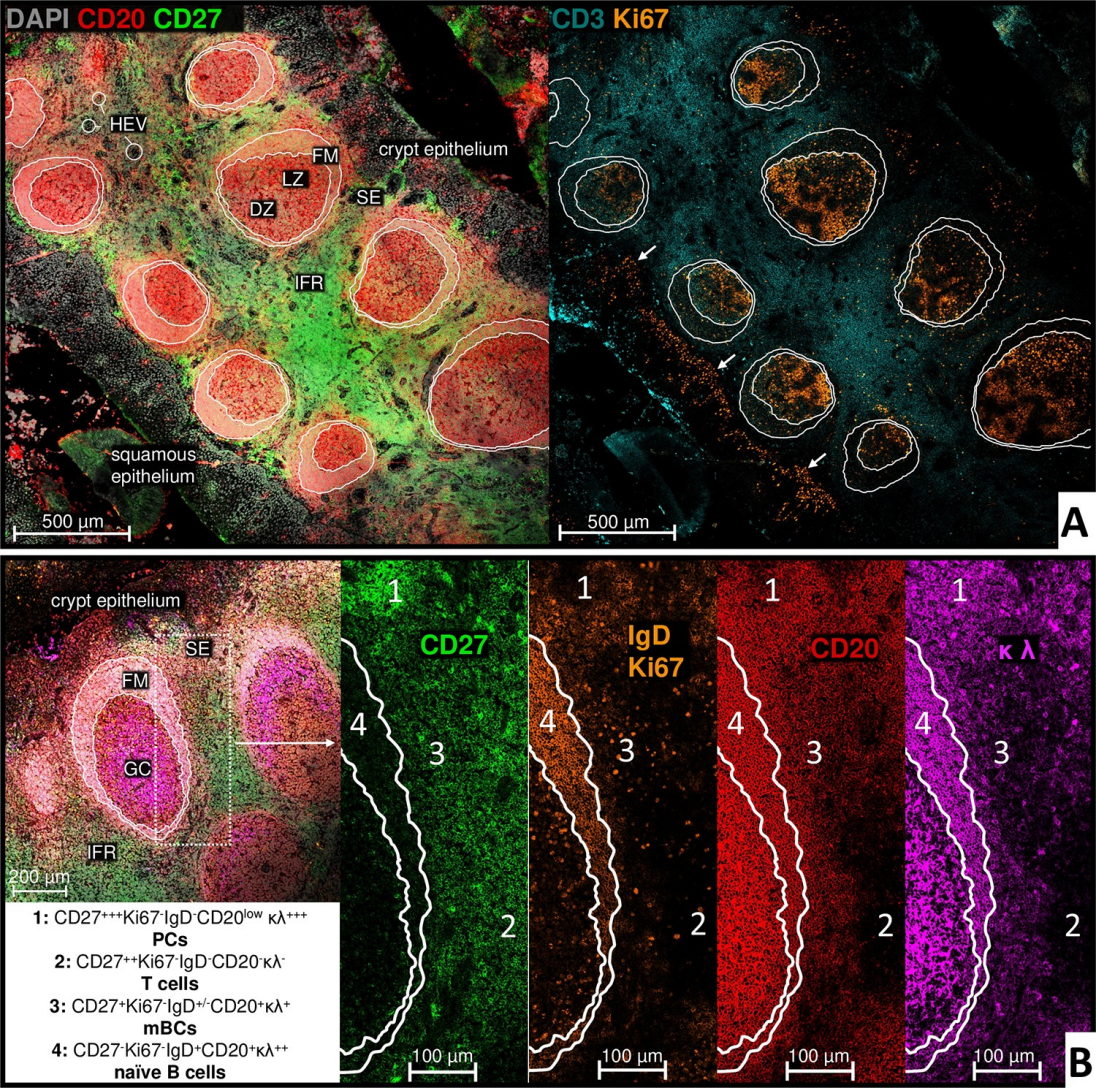
Expression of CD27 defines distinct areas in the tonsil. White lines within and around the follicle are drawn for better orientation of follicular zones in the different channels. **A: Overview of lymphocyte distribution in the human palatine tonsil, representative example.** [Human tonsil; 10x objective]: Asymmetry of Ki67 rich DZ directed to the CD3+ IFR and a broadening FM directed to the SE. Crypt epithelium with a loose cell structure, many CD20+ B cells and only few Ki67+ proliferating cells compared to the squamous epithelium identified via Ki67+ cell rich area above basal lamina (white arrows). Superficial follicular CD27+CD20+Ki67- mBCs can already be displayed in this resolution by a mixed signal of CD27 and CD20 channel. **B: Differentiation of lymphocyte populations according to their CD27 expression.** [Human tonsil; 20x objective]: Four different CD27-FITC signal intensity zones (1 to 4) could be identified and in combination with Ki67, IgD, CD20 and κλ expression resulting main populations are indicated in the textbox (lower left).

Subsequent studies examined the organization of mBCs in human palatine tonsils. This is the first detailed histological description of CD27 expressing tonsillar B and T lymphocyte localization. The following section will address the evaluated subpopulations by assessing the expression intensity. All 15 tonsil specimens exhibited multiple asymmetrical secondary follicles with DZ and LZ, with an average diameter twice as large as found in splenic secondary follicles (Fig 4A). Ten tonsils exhibited GCs that appeared to dissolve with numerous core fragments and lifted tissue architecture (S1A Fig). Six tonsils showed kidney-shaped (S1B Fig) and hourglass-shaped constricted follicular formations as well as a perforated FM or one GC within another, which could not be explained by cutting. Comparable to the spleen, no primary follicles were detected in any of the investigated tonsil sample sections. Of note, MAdCAM-1 was not expressed by any of the investigated specimens.

#### Extrafollicular CD27+++Ki67-IgD+/-CD20(low)κλ+++PCs

**Fig 5:**
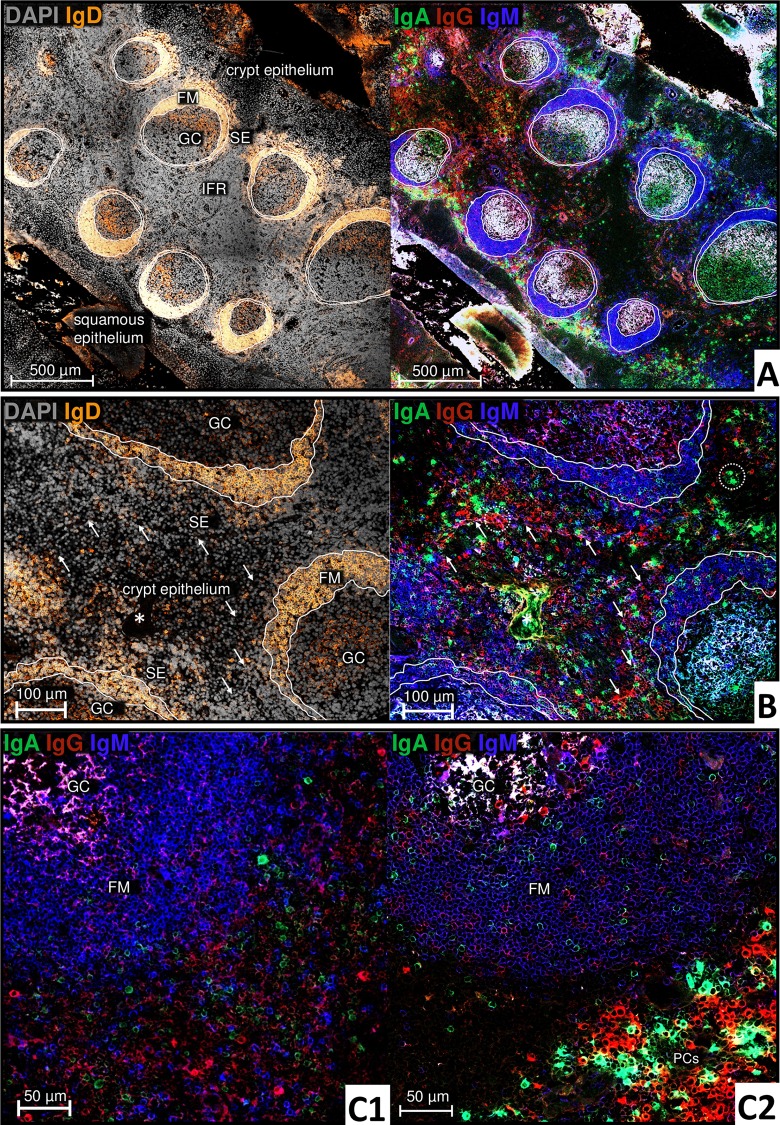
Switched tonsillar mBCs are heterogeneously distributed in the superficial zone and the FM. White lines within and around the follicle are drawn for better orientation of follicular are drawn zones in the different channels. **A:** [Human tonsil; 10x objective] Serial section corresponding to Fig 4A. FM with mainly IgD+ naïve B cells. Bright extrafollicular intracellular IgG and IgA signal by PCs mainly in the SE, but also superficial follicular, in the IFR and the crypt epithelium. IgA+ or IgG+ and IgM+IgD+ mBCs, respectively, cannot be adequately resolved at this resolution; lower specific Ig signals can be identified primarily superficial follicular and in the FM. Unspecific bindings with vascular connective tissue especially in the IFR. Within the GC, intercellular signals may arise from immune complexes of the resident follicular dendritic cells. **B:** [Human tonsil; 20x objective]: White arrows mark the transition from crypt epithelium to SE. Bright extrafollicular intracellular IgG and IgA signal by PCs (exemplified in white circles). Heterogeneously distributed IgG+ and IgA+ cells in the SE, FM and epithelium. * Crypt lumen with detritus. **C:** [C1 human tonsil; 20x objective, C2 human tonsil; 63x objective]: Switched IgA+ and IgG+ mBCs are heterogeneously distributed in the superficial follicular zone (C1) and the FM (C2).

In the tonsil, the strongest CD27 signal was detected on PCs (Fig 4B, area 1). Moreover, they could be well morphologically identified since they appeared with a large immunoglobulin containing cytoplasm. Accumulations of PCs were found mainly in large clusters in the SE of all specimens. PCs in the IFR were either arranged as clusters around vascular lumen or forming lines with decreasing number towards the tonsillar center. The different specimens showed a high variability in PC frequency, ranging from some cells located mainly in the SE to large accumulations distributed over the entire tissue. In 14 out of the 15 investigated samples, IgA+ and IgG+ PCs were observed to be the main phenotype (Fig 5), whereas IgD+ PCs were only occasionally found. However, one tonsil showed a clearly increased number of IgD+ PCs. In all tonsils, the few IgM+ PCs found populated mainly the SE.

#### Extrafollicular CD27++ Ki67-IgD-CD20-κλ- T cells

As in spleens, tonsillar T cells showed an increased CD27 signal compared to mBCs (Fig 4B, area 2). The main population was located in the IFR with increasing frequency towards the tonsillar center. Some Ki67+ proliferating T cells occurred at the transition to the follicular area.

#### Extrafollicular CD27+ Ki67- IgD+/-CD20+κλ+ mBCs

In the SE and superficial follicular area, a variable mBC compartment was found to form a patchy hem around the entire secondary follicle in all specimens (Fig 4B, area 3). The irregular mBC pattern widened towards the epithelium with a maximum of cells in the SE. In contrast to the spleen, this area appeared to be variable within and between the different specimens, from very clear to very thin or incomplete. A broad and symmetrical structural organization similar to that of the splenic MZ could not be detected, but the general property of follicular association and irregular borders with a gradual decline of mBCs towards the FM was identical. Albeit rare, in two tonsils remarkably extensive mBC areas were found in the SE and around the follicle with very large GCs and relatively narrow FMs. Compared to PCs and naïve B cells, the Ig staining signal on mBCs was found less intense. SE and superficial follicular IgA+ and IgG+ post-switch mBCs were always detected heterogeneously distributed in small clusters of only a few cells each and with a tendency of increasing number in distance to the follicle compared to pre-switched IgM+IgD+ mBCs (Fig 5B). Additionally and similar as in the spleen, individual IgG+ or IgA+ mBC were also identified in the FM (Fig 5C2).

#### Intrafollicular CD27-Ki67-IgD+CD20+κλ++ naïve B cells and other CD27+/- lymphocytes

The follicular area (GC and FM) within tonsils showed a diminished signal for CD27 compared to the rest of the tissue analyzed (Fig 4B, area 4, FM), except for Ki67+ plasmablasts and T cells populating the transition between FM and GC. The Ig signal of individual cells within the GC was superimposed since this area contains immune complexes (IC) with follicular dendritic cells especially in the LZ. In all specimens, pre- and post-switched mBCs populated the FM alongside the main naïve B cell population. Interestingly, four specimens included follicles that contained GCs with either only IgA or IgG expressing B-cells, respectively.

## Discussion

The present study reports on the successful establishment of immunofluorescence multicolor stainings using primary and secondary antibodies in a hapten and signal amplification system on frozen unfixed and fixed human spleen and tonsil tissue. As a result, we were able to use several mouse antibodies in parallel, which allows visualization of corresponding flow cytometric generated data [5] by using the same antibodies and allowing better comparison to the primarily multicolor immunofluorescence histology in mice studies. On the other hand, this corresponding flow cytometric data allowed to determine absolute percentage distributions of the observed cell populations. Since isolated tissue sections lack a three-dimensional information and distribution complexities, we found such calculations to be not expedient here.

The simultaneous but separate digital information of five fluorescence parameters enabled us to originally characterize the microenvironment of human Ki67-CD27+CD20+ mBCs within one frozen section. In our experience, the overlapping excitation and emission spectra make conventional LSM data of more than 5–6 parallel channels difficult to unmix with the given instrumentation. More detailed analysis with >6 marker information per section could be obtained by new very complex methods like imaging mass cytometry and imaging cycler microscopy technologies. [18] These methods would, however, not directly visualize corresponding flow cytometric data because of the different underlying technical principles. Furthermore, equivalent resolution of image data from LSM (nanometer range) cannot be achieved by imaging mass cytometry so far. [19]

### Human organ-specific mBC compartments or generally follicle associated mBC areas?

As a common feature of human splenic secondary follicles, demonstrated by us in spleen specimens S8 and S9 as well as observed in all investigated tonsil specimens T1 –T15, the predominating mBC population appears as follicular structure with a mixture of mBCs and naïve B cells in transition to FM. In contrast, the marginal sinus in mice and rats clearly discriminates between these zones in spleen and furthermore, this separated MZ surrounds the entire white pulp complex. [9, 13, 20] In human spleen, we found naïve B cells around the PALS and on the border from oMZ into the perifollicular and red pulp area as surrounding compartment of the white pulp. The absence of such naïve B cells at the outermost mBC area in tonsils might be related to different migration pathways. Lymph nodes and MALTs are migrated to via high endothelial venules (HEV), which do not exist in the spleen. Detailed mechanistic studies on human mBC recirculation and migration are missing so far, but since not only anatomical differences with rodents indicate that they behave differently [9–11], renaming the human MZ would prevent incorrect extrapolation. Since rodents lack palatine tonsils [21], further and direct comparison with this SLO was not possible.

Steiniger et al. [13] studied the distribution of CD27+CD3- human mBCs on paraffin sections where they showed that CD27+ B cells can colonize the entire follicular periphery around the GC in all investigated SLOs. In contrast to symmetrical splenic MZ, the CD27+ B cell area in lymph nodes, appendices and terminal ileum was always found to be asymmetrical and widening towards the epithelium. However, when applied to tonsils, subtraction staining failed to define a CD27+CD3- superficial follicular area for the majority of specimens. Our results provide the missing data in this regard and further support the data by Steiniger et al. [13]. The hypothesis of a generally superficial follicular arranged mBC area in humans with structural variability would thus be in accordance with the available data. A recent imaging mass cytometry analysis of gut lymphoid tissues observed CD27+IgM-IgD- switched mBCs located on the periphery of the entire tissue and surrounding a CD27+IgM+IgD+ as MZ population defined B cell accumulation, which were more confined distributed between the GC and the follicle-associated epithelium. [14] This study also interrogated spleen and tonsil with regard to the developmental relationship between classical mBC subsets and MZ mBC and concluded that they are not developmentally contiguous, however, more data is needed to reach a final conclusion. It would be very interesting to have data sets of other lymphoid tissues with this technology combined with repertoire sequencing.

The existence of a human MZ is still subject of controversies and there are multiple indications that the majority of rodent organ specific MZ B cells differ from human mBCs in terms of their function, mutation state as well as migratory behavior. [7–11] By contrast, analyses of MZ defined micro-dissected B cells of follicle surrounding areas in human spleen [22], Payer’s patches [23] and lymph nodes [24] found mixed B-cell populations sharing phenotypic and molecular characteristics, such as hypermutation. Flow cytometric analysis showed that phenotypically similar human mBCs are distributed over lymphatic organs including the spleen as well as the peripheral blood [5], which together with recent findings about large clonal repertoire overlaps between these tissues [6] excludes that these cells reside exclusively in the human spleen. The rodent organ-specific MZ might be species specific. The assumption of an obligate follicle associated mBC area would also be consistent with the idea of the systemic recirculation of human MZ mBCs, the latter which is supported by the effective depletion of all splenic CD20+ B cell subsets by rituximab [25, 26]. Others already favored the idea of a dynamic B-cell compartment since human reactive lymph nodes contained MZ lymphocytes. [27]

Based on the present work, we support the hypothesis of a superficial follicular organized mBC area with universal properties but structural variability consistent with earlier proposals [9, 11], and to rename the human splenic MZ ‘superficial zone’ to allow a better differentiation. The application of our method should be independently validated in additional spleen specimens.

### Differences in superficial follicular mBC area symmetries—A correlate of follicular stage?

Surgical indication for tonsillectomy of the here analyzed tonsils was tonsillitis with substantial inflammation outside an acute flare, which might cause multiple GC reactions, as documented. Spleens included for MZ evaluation (S8 and S9) were not primarily influenced by immunological disease patterns and since secondary follicles with degenerating GCs represent the typical stage of human spleen in adulthood [28], it was not surprising that we did not find as many reactive follicles with clear LZ and DZ as in tonsil. However, the analysis of a trauma spleen from a 6-year-old showed pronounced asymmetric secondary follicles and a surrounding asymmetrical mBC area widening with distance from the PALS [13], i.e. T cell zone, as we described here for spleen and tonsil. Interestingly and in contrast to all studied tonsils, splenic follicles of the same donor appeared to be in a similar development status. Depending on local antigen supply and immunological status, follicles may persist and be reactivated or replaced in adaptation to changes in antigen supply. Tonsils are constantly in direct antigen contact from the oral cavity via their crypt epithelium, the spleen in contrast is only supplied by antigen via the blood. High antigen doses can cause apoptosis of GC B cells [29], what might explain the observed tonsillar GCs, which seemed to dissolve or collapse while Ki67+ B cells were still present. Recent studies reported tremendous variation and flexibility in GC function [30], but detailed models on how follicular formations in long-term and context depending proceed periodically or alternate and regress are missing so far. It remains an open question whether the differences in superficial follicular mBC area symmetries are a correlate of an organ-specific mBC organization or simply the consequence of a different follicular status.

### Differences in the human splenic versus tonsillar pre-switch—Post-switch mBC proportion and the importance of the human splenic MZ

The density of IgM-IgD- post-switch mBCs increased towards the direction of the red pulp but was generally diminished compared to IgM+IgD+/- mBCs. However, since the cells are very close to each other in direction to the FM, the use of an indirect definition appears to be less reliable. We analyzed the four predominating mBC Ig isotypes for the first time within one frozen section. In agreement with serial paraffin sections each stained for IgM, IgD and IgA by Steiniger [9], respectively, we found a trend of a red pulp-directed increase of post-switched mBCs, which contained IgA or IgG mBC at various degrees. The ratio of these isotypes was heterogeneous and lacked any dominance, similar to results of a study of frozen sections for human splenic follicles [31]. To the best of our knowledge, human tonsillar mBC subsets have not yet been histologically examined in such detail. We observed a similar isotype distribution without clusters and an increase of the post-switch mBC population towards the outermost superficial follicular mBC area. In agreement with flow cytometry data [5], considerably fewer IgM+IgD+/- mBC were detected in tonsils in contrast to spleens. A relative accumulation of post-switch mBCs in tonsils could result from their function as MALT, by which environmental mucosal epithelia and exocrine glands are coated with a layer of antimicrobial IgA antibodies. [21] Notably, however, our study found also a substantial presence of IgG+ mBC in tonsils similar as for spleens. Although the underlying main specificities may differ, the overall data support that both isotypes are comparably distributed and suggest their relevance for humoral mucosal and systemic memory.

The role of the human splenic MZ for recirculating IgM+IgD+/- MZ mBCs is not fully understood but appears to depend on the presence of the spleen. [9] Splenectomized individuals have been considered to carry higher risks of infection with encapsulated bacteria due to impaired production of IgM+IgD+ MZ B cells against polysaccharides. [32] As alternative explanation, this risk can also be at least partially explained by removal of the red pulp, one of the largest phagocytosing compartment of the human body. MZ B cell defects have been documented less severe in children [33] and a normal MZ B cell population was also found in the blood of children with congenital asplenia, so that MZ B cell precursors may colonize alternative areas [34]. Furthermore, studies indicate a functional relation between spleen and GALT. [35–37] It has been suggested that human MZ B cells are derived from transitional B cells activated in GALT, which may explain the presence of mutations and their specific direction against bacterial antigens. [38] The fact that an exchange of MZ and intestinal B cells can basically occur is reflected by the observation that MALT lymphomas frequently colonize the splenic MZ. [39] This would be consistent with the integrin beta 7 expression on the majority of human splenic mBCs [5] and the expression of its homing ligand MAdCAM-1 in the human splenic MZ and Peyer’s patches but not in tonsils [40].

In summary, we here show for the first time an innovative five color staining of human lymphoid tissue sections applicable for native antigen detection. The data on mBC aggregation support to rename the human splenic marginal zone to ‘superficial zone‘ that also applies to the human palatine tonsil. Regarding the observed differences in mBC area symmetries including their Ig isotype proportion, the significance of B cells interacting with follicular structures for immune memory require further consideration.

## Supporting information

S1 Fig Diversity of tonsillar secondary follicles. [A and B: sections of human tonsil; 20x objective]. **A:** Secondary follicle that appears to dissolve with lifted tissue architecture (exemplarily marked in a circle) and perforated FM with complete interruptions (arrows). * Crypt lumen. **B:** Kidney-shaped constricted follicular formation (GC 1 / FM 1), which could not be explained by cutting. Follicle GC 2 / FM 2 might be cut like marked by the white dotted line.
(PDF)
