# Supplementary figures and images for: Human CD27+ memory B cells colonize a superficial follicular zone in the palatine tonsils with similarities to the spleen. A multicolor immunofluorescence study of lymphoid tissue
Source: PLoS One. 2020 Mar 18;15(3):e0229778. doi: 10.1371/journal.pone.0229778 (PMC7080255; doi:10.1371/journal.pone.0229778)

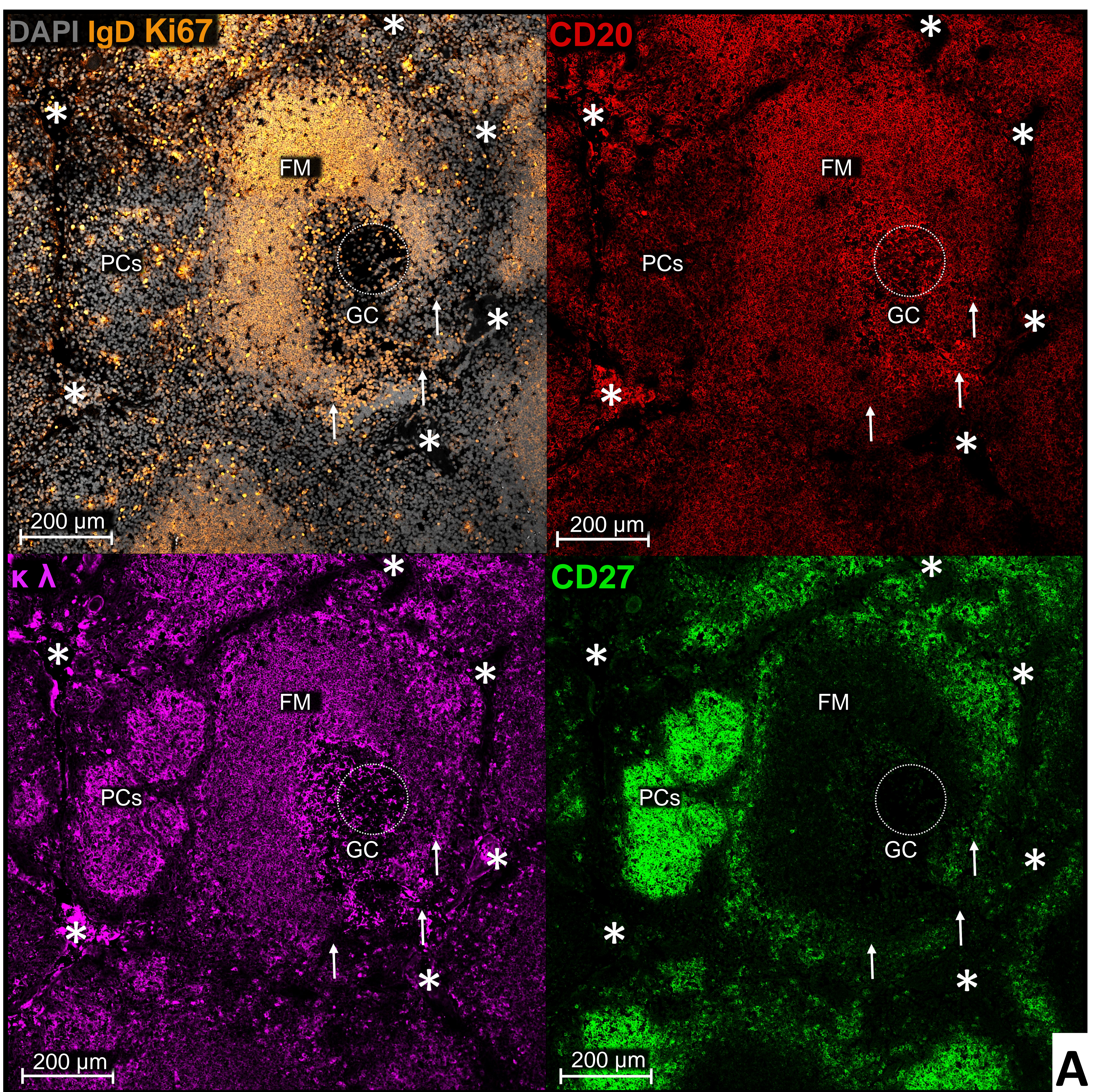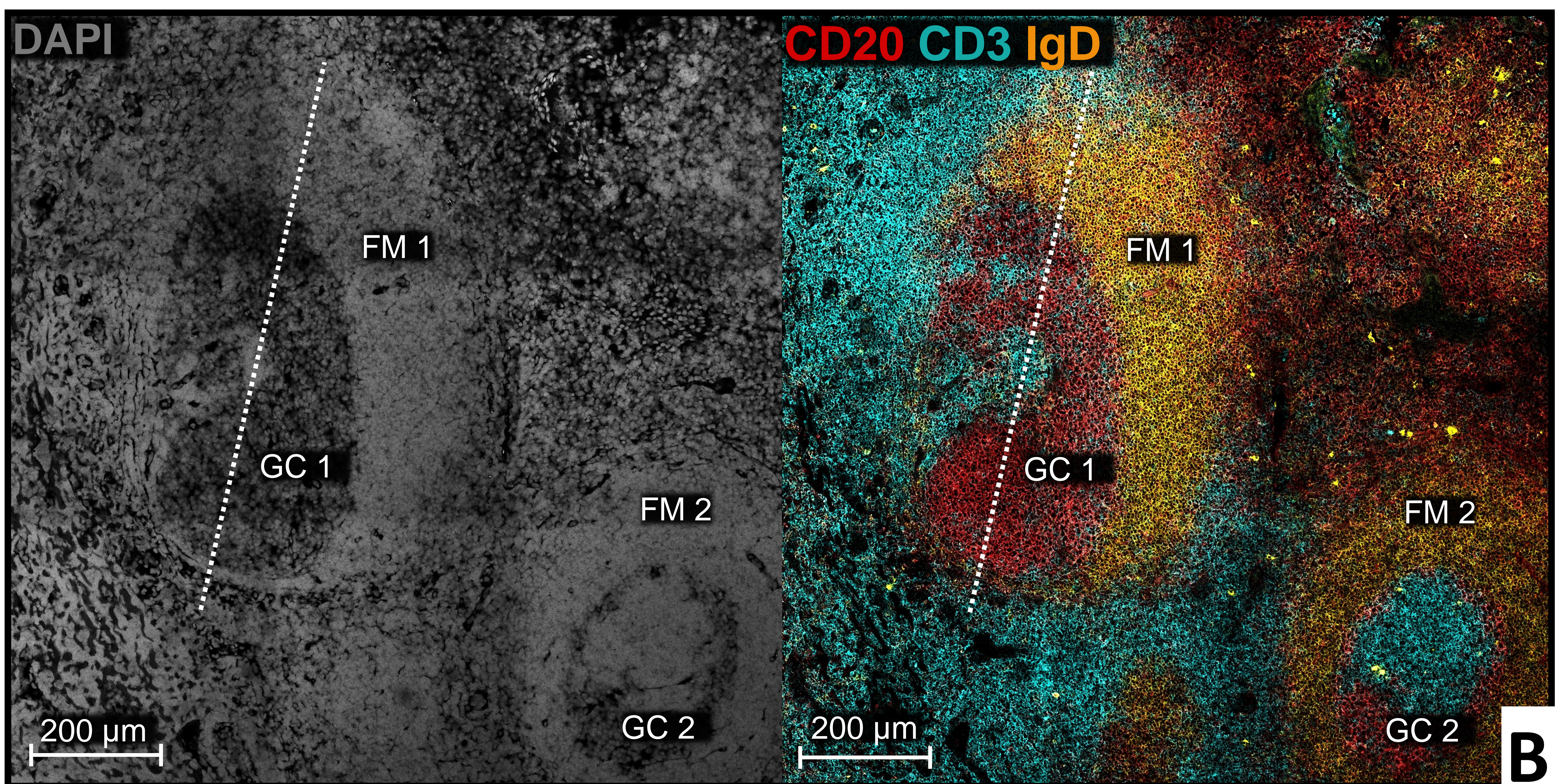

Supplement: S1 Fig — [A and B: sections of human tonsil; 20x objective]. A: Secondary follicle that appears to dissolve with lifted tissue architecture (exemplarily marked in a circle) and perforated FM with complete interruptions (arrows). * Crypt lumen. B: Kidney-shaped constricted follicular formation (GC 1 / FM 1), which could not be explained by cutting. Follicle GC 2 / FM 2 might be cut like marked by the white dotted line. (PDF) [file pone.0229778.s001.pdf]
